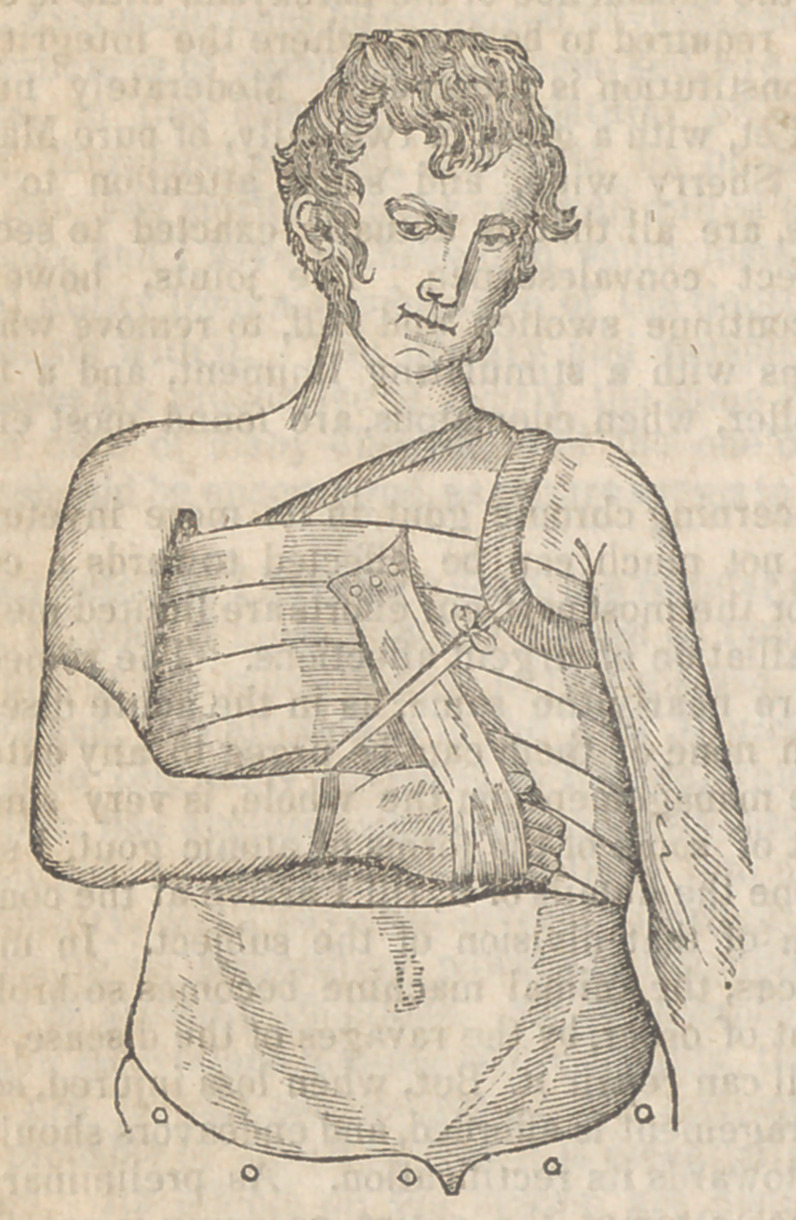# Description of an Apparatus for the Treatment of Fractures of the Clavicle

**Published:** 1838-03-28

**Authors:** George Fox

**Affiliations:** Surgeon to the Wills’ Hospital, &c.


*Description of an apparatus for the treatment of Fractures of the Clavicle.* By GEORGE FOX, M. D. Surgeon to the Wills' Hospital, &c.

This apparatus consists of a pad, a roller, an elbow piece, a loop, a sling and a broad band.

The *pad* (which is placed in the axilla of the injured side) should be no larger than sufficient to fill the natural vacuity between the arm and the side of the chest, wedged shaped, rather thicker and larger at the end which is placed in the axilla than below. This part is secured by a *roller* passing around on the thorax, and if thought requisite by tapes passing over the shoulder of the opposite side, and tied in the axilla.

The *elbow-piece*, is a sling, adapted to the size and shape of the elbow, when the fore arm is placed at right angles to the arm, open throughout its whole extent; it extends about half way up the arm and two thirds the way down the fore arm; to the centre of the upper end, a band, one to two feet long, (a piece of broad tape answers extremely well) is attached; tapes are also fixed to each corner of the other end; it may be made of any material which can be conveniently procured at the time, muslin or linen wadded with cotton is preferable.

A circular *loop* or collar, made of linen filled with cotton, is placed over the shoulder of the sound side, to which the band from the upper end of the elbow piece is affixed behind, and the tapes from the lower portion in front.

A small *sling* for the hand is attached to the roller which secures the pad.

Over the whole a *broad band* of muslin, extending from within one and a half to two inches of the top of the shoulder, to the elbow, is then applied, this band is sometimes dispensed with, but answers the purpose of fixing the elbow, consequently keeping the shoulder outwards, and prevents the patient from interfering with the apparatus; to make it more comfortable it may be wadded.

The apparatus will be readily understood by reference to the accompanying drawing, it is simple, can be procured as easily and in as short time as Desault's, and is thought to possess considerable advantages over it, among which we may mention, that it is much more comfortable to the patient, especially in the case of females; is of much easier application and can be more readily adjusted, and that without disturbing the fracture; it enables the surgeon to detect in an instant any displacement which may have occurred, the seat of injury being in most cases exposed to view. I say in most instances, for cases occasionally occur, in which from the action of the muscles inserted into the clavicle, there is a strong tendency to displacement, one fractured end being drawn upwards; this can be corrected by the application of a compress to the part, secured by two or three turns of a roller over it and around the elbow of the injured side. The *pad* for the axilla being much smaller than Desault's is therefore less liable, from pressure on the axillary plexus, to produce paralysis of the arm. We are well satisfied that this apparatus is better adapted than Desault's to the treatment of most, if not all injuries of the clavicle. Many cases of perfect cures, without the *slightest deformity* have been accomplished by its use. This result is contrary to the opinion of many surgeons who say that this accident is always followed by deformity.

The apparatus just described was introduced into the practice of the Pennsylvania Hospital in 1828, for the treatment of fractures and dislocations of the clavicle, and as remarked by Dr. Wallace in his "Statistical account of Fractures," published in the second number of the Examiner, "has since been used to the exclusion of other methods of treatment;" its modifications were chiefly made at the suggestion of my friend Dr. James A. Washington, of New York, then my colleague in that institution.

Philadelphia, Feb. 27th, 1838.

## Figures and Tables

**Figure f1:**